# Recurrent Wheeze Exacerbations Following Acute Bronchiolitis—A Machine Learning Approach

**DOI:** 10.3389/falgy.2021.728389

**Published:** 2021-11-02

**Authors:** Heidi Makrinioti, Paraskevi Maggina, John Lakoumentas, Paraskevi Xepapadaki, Stella Taka, Spyridon Megremis, Maria Manioudaki, Sebastian L. Johnston, Maria Tsolia, Vassiliki Papaevangelou, Nikolaos G. Papadopoulos

**Affiliations:** ^1^West Middlesex University Hospital, Chelsea and Westminster Foundation Trust, Isleworth, United Kingdom; ^2^Centre for Paediatrics and Child Health, Imperial College London, London, United Kingdom; ^3^Allergy and Clinical Immunology Laboratory, Second Department of Pediatrics, National and Kapodistrian University of Athens (NKUA), School of Medicine, P. and A. Kyriakou Children's Hospital, Athens, Greece; ^4^Division of Evolution, Infection and Genomics, University of Manchester, Manchester, United Kingdom; ^5^National Heart and Lung Institute, Imperial College London, London, United Kingdom; ^6^Second Department of Pediatrics, National and Kapodistrian University of Athens (NKUA), School of Medicine, P. and A. Kyriakou Children's Hospital, Athens, Greece; ^7^Third Department of Paediatrics, Attikon University General Hospital, School of Medicine, National and Kapodistrian University of Athens, Athens, Greece; ^8^Division of Infection, Immunity and Respiratory Medicine, University of Manchester, Manchester, United Kingdom

**Keywords:** bronchiolitis, wheeze, virus, rhinovirus, machine learning

## Abstract

**Introduction:** Acute bronchiolitis is one of the most common respiratory infections in infancy. Although most infants with bronchiolitis do not get hospitalized, infants with hospitalized bronchiolitis are more likely to develop wheeze exacerbations during the first years of life. The objective of this prospective cohort study was to develop machine learning models to predict incidence and persistence of wheeze exacerbations following the first hospitalized episode of acute bronchiolitis.

**Methods:** One hundred thirty-one otherwise healthy term infants hospitalized with the first episode of bronchiolitis at a tertiary pediatric hospital in Athens, Greece, and 73 age-matched controls were recruited. All patients/controls were followed up for 3 years with 6-monthly telephone reviews. Through principal component analysis (PCA), a cluster model was used to describe main outcomes. Associations between virus type and the clusters and between virus type and other clinical characteristics and demographic data were identified. Through random forest classification, a prediction model with smallest classification error was identified. Primary outcomes included the incidence and the number of caregiver-reported wheeze exacerbations.

**Results:** PCA identified 2 clusters of the outcome measures (Cluster 1 and Cluster 2) that were significantly associated with the number of recurrent wheeze episodes over 3-years of follow-up (Chi-Squared, *p* < 0.001). Cluster 1 included infants who presented higher number of wheeze exacerbations over follow-up time. Rhinovirus (RV) detection was more common in Cluster 1 and was more strongly associated with clinical severity on admission (*p* < 0.01). A prediction model based on virus type and clinical severity could predict Cluster 1 with an overall error 0.1145 (sensitivity 75.56% and specificity 91.86%).

**Conclusion:** A prediction model based on virus type and clinical severity of first hospitalized episode of bronchiolitis could predict sensitively the incidence and persistence of wheeze exacerbations during a 3-year follow-up. Virus type (RV) was the strongest predictor.

## Introduction

Acute bronchiolitis is a common respiratory condition in infancy. Although most infants with bronchiolitis are managed in primary care, the number of hospitalizations and associated complications remains high (1). This condition impacts not only on healthcare resources' utilization, but also on caregivers' anxiety and on family quality time (2).

The epidemiology of bronchiolitis varies from country to country and with season (3). Although Respiratory Syncytial Virus (RSV) is considered the main trigger, other viral agents are also frequently detected (4–6). Rhinoviruses (RVs), human metapneumovirus (MPV), influenza (IFV), parainfluenza viruses (PIVs), adenoviruses, coronaviruses and human bocavirus can be detected both as a single and as co-infective agents (7).

Not all episodes of hospitalized bronchiolitis are severe. The severity of episodes of hospitalized bronchiolitis ranges from moderate, where infants just require observation and supportive therapies (supplemental oxygen, fluids, and nutrition) to high severity, where infants can require intensive care admission and/or die (8). Observational studies have identified risk factors for higher severity of bronchiolitis, such as age, prematurity, chronic lung disease and viral etiology. RSV has been considered as the trigger of more severe episodes of acute bronchiolitis. Some case control studies have associated RV, MPV and other viruses with increased clinical severity as well (9). The role of multiple viral agents in bronchiolitis pathogenesis has been repeatedly reported, with up to 61% of infants with bronchiolitis testing positive for multiple viruses (7). Viral co-infections have also been associated with increased disease severity and prolonged hospital stay (10).

Severe bronchiolitis in infancy has been associated with increased incidence of preschool wheeze exacerbations and with asthma diagnosis at school years (11, 12). Interestingly, specific viruses have been associated with differential risk for persistent wheeze and asthma (13). RSV-bronchiolitis was most associated with recurrent wheeze exacerbations during the first 3 years of life (14). These reported associations have been mainly based on comparisons between RSV-bronchiolitis and healthy controls (15). There is ongoing evidence associating RV-bronchiolitis with higher number of recurrent wheeze exacerbations in preschool years when comparing with RSV-bronchiolitis (16, 17). The mechanisms underlying these associations are still not clearly understood.

It has been proposed that RSV-bronchiolitis can cause airway damage that promotes smaller airway obstruction and wheeze (18). On the other hand, RV likely causes less structural damage but is a potent trigger of airway hyper-reactivity (18). Rather than sole viral etiology, interactions with host immune response to viral triggers, personal risk factors, and environmental exposures have been proposed to underly a complex recurrent wheeze development pathway (13).

In this study, we focused mainly on interactions between respiratory virus type and clinical characteristics, and we hypothesized that specific respiratory virus, and other (demographic and clinical characteristics) factors could consist predictors of recurrent wheeze exacerbations following the first episode of hospitalized bronchiolitis. We performed principal component analysis to identify clusters of persistence of wheeze following bronchiolitis hospitalization. We based our prediction model on the cluster that was associated with the highest incidence of wheeze exacerbations during the 3-year follow-up period. Finally, we utilized machine learning methodology to identify the strongest predictors of this cluster.

## Methods

### Study Design

This was a prospective, single-center cohort study of infants hospitalized with first episode of bronchiolitis and were followed up for 3 years. Approval was received from the Institutional Ethics Committee. The parents of all infants provided written informed consent at the time of enrollment.

**Figure 1:**
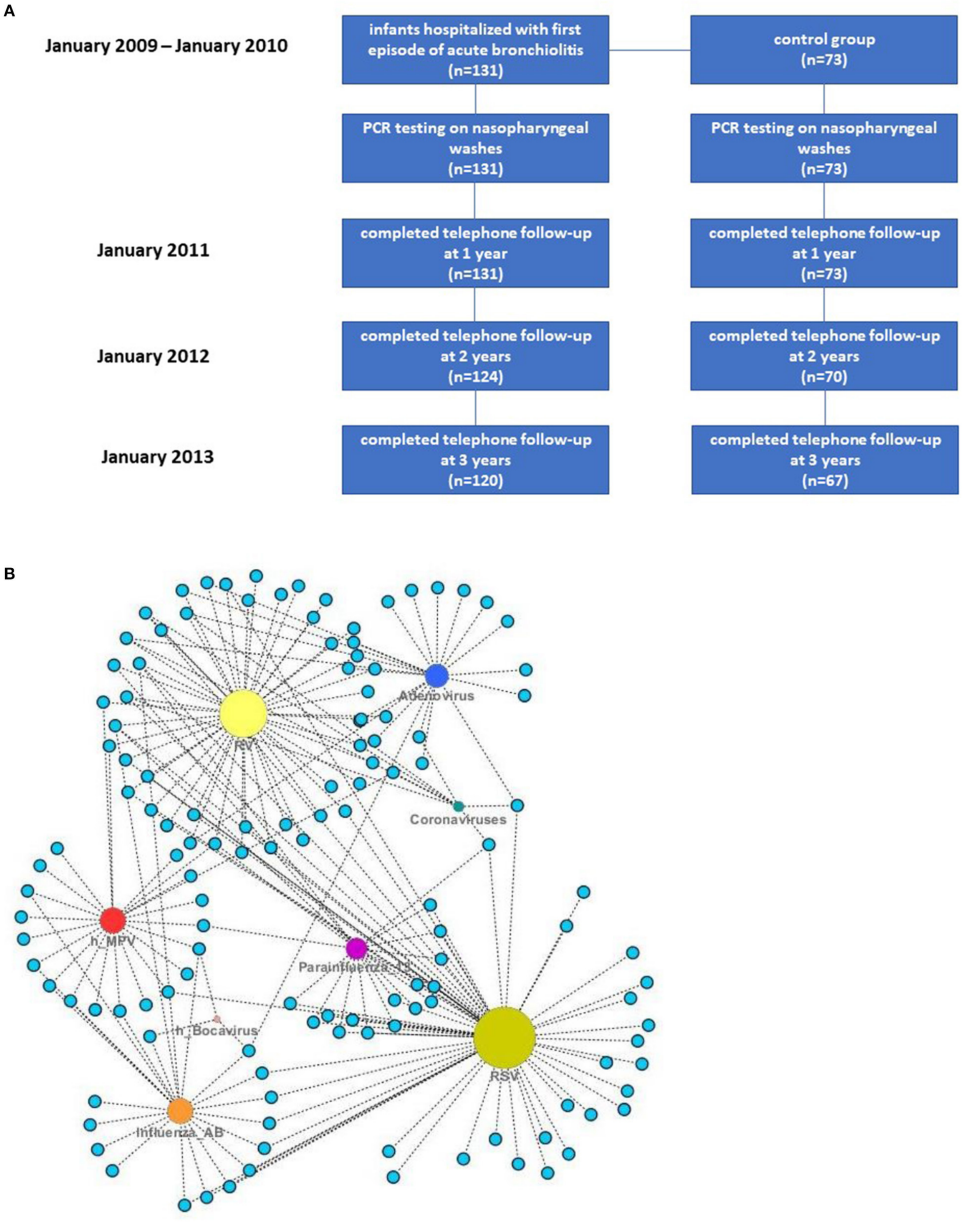
**(A)** Flow diagram describing patients' recruitment and follow-up. **(B)** Absolute frequencies of viruses detected in nasopharyngeal washes of infants with bronchiolitis and control patients—each node represents a patient and size is related to the frequency of detected viral agents.

A total of 131 otherwise healthy term infants (under 18 months old) hospitalized for their first episode of acute bronchiolitis were recruited. Seventy three infants (under 18 months old) who presented to the emergency department (2nd Pediatric Clinic, University of Athens) due to a minor accident or due to a scheduled visit for surgical review (inguinal hernia, hydrocele) were recruited as controls. The recruitment period lasted over 2 years (December 2008 till December 2010). All infants were prospectively followed up for 3 years following initial admission (completed in January 2013). The flowchart describing the recruitment and follow up is set on Figure 1A.

Acute bronchiolitis was diagnosed by the attending pediatrician as an acute respiratory illness with some combination of rhinitis, cough, tachypnoea, wheezing, crackles, and signs of respiratory distress, fever or no fever in infants younger than 2 years old (19). The severity of the episode of acute bronchiolitis was assessed based on a previously validated score that assesses heart rate, respiratory rate, oxygen saturation, wheeze and feeding difficulty (20). This score ranges between 5 and 15 (mild to increased disease severity).

### Data Collection

The research team conducted a structured interview that assessed patients' demographic characteristics, medical and environmental history, and details of the clinical presentation. Daily hospital medical records provided further clinical data until discharge from hospital. Nasopharyngeal washes were collected from infants with bronchiolitis and controls and a panel of 8 respiratory viruses was assessed by qualitative polymerase chain reaction (PCR). This panel included RSV, RV, adenovirus, MPV, human coronavirus (HKU1, NL-63), IFV A and B, PIV 1-3 and human bocavirus (21).

### Statistical Analysis

The “cluster group” was generated by the 6-monthly wheeze episode via agglomerative, hierarchical, and using Ward's linkage method data clustering. The optimal number of clusters was defined following the “majority rule” and yielded to number 2. Thus, the “cluster group” was labeled as “1,” or “2” (http://www.jstatsoft.org/). Average wheeze episodes per cluster group were plotted with time (Supplementary Figure 1A).

Through principal component analysis (PCA), the individuals were plotted on the Euclidean space of the two first principal components occurring from the wheeze episodes data (Supplementary Figure 1B).

Independent variables (demographics, clinical characteristics, virus type) were examined for association with the dependent variables (Clusters 1 and 2). Quantitative predictors were checked for normality with the Shapiro-Wilk test, and non-parametric tests were applied in non-normal parameters. Wilcoxon's rank-sum test and Kruskal-Wallis tests were applied for quantitative parameters, and descriptive statistics were provided in the form of a median (25th−75th percentile). Pearson's Chi-Squared test of independence was performed for qualitative parameters.

To detect the significant predictors for Cluster 1 (the worst outcome), multi-factorial analysis by using a random forest classifier was performed. The prediction model assessed prediction sensitivity and specificity of the clusters from independent variables and virus groups. Prior to modeling, missing values were imputed with a random forest-based algorithm (22).

All statistical tests were considered as two-sided, and statistical significance was taken when *p* < 0.05. Statistical analysis was conducted via the R software for statistical computing.

## Results

Demographic and clinical characteristics and virology data of infants hospitalized with bronchiolitis are presented below.

### Demographics—Clinical Characteristics-Virology Data

#### Demographics

Demographic characteristics of infants with bronchiolitis are depicted on Table 1.

**Table 1:** Demographic characteristics of infants with bronchiolitis.

**Demographics**	**Bronchiolitis infants**
**Age (months)**	
Median (Q_1_-Q_2_)	2.67 (1.25, 4)
**Gender**, ***n*** **(%)**	
Male	75 (57.3%)
Female	56 (42.7%)
**Birth weight (Kg)**	
Median (Q_1_-Q_2_)	3.15 (2.83–3.4)
**Breast feeding duration (days)**	
Median (Q_1_-Q_2_)	30 (14–60)
**Type of delivery**	
Normal delivery	69 (53%)
Cesarean section	62 (47%)
**Maternal smoking during pregnancy**	
*n* (%)	56 (42.7%)
**No of children having brothers/sisters**	
*n* (%)	75 (57.3%)
**Allergies in parents**	
Mother, *n* (%)	19 (14.5%)
Father, *n* (%)	23 (17.6%)
**Allergies in brothers/sisters**	
*n* (%)	18 (13.7%)
**Asthma in parents**	
Mother, *n* (%)	8 (6.1%)
Father, *n* (%)	12 (9.2%)
**Asthma in brothers/sisters**	
*n* (%)	12 (9.2%)
**Eczema presence**	
*n* (%)	25 (19.1%)
**Food allergies**	
*n* (%)	8 (6.1%)

#### Virus Detection

Absolute frequencies of virus detection in cases and controls are presented on Table 2. In 57 out of 131 infants with bronchiolitis (43.5%) there was more than one virus detected in nasopharyngeal washes. Viruses detected as single and/or multiple pathogens are depicted at Figure 1B.

**Table 2:** Absolute frequencies of viruses detected in nasopharyngeal washes of infants with bronchiolitis.

**Respiratory viruses**	***n*** **(%)**
RSV	58 (44.3%)
RV	44 (33.6%)
Adenovirus	21 (16%)
MPV	22 (16.8%)
Influenza virus (IFV)	21 (16%)
Parainfluenza virus (PIV)	18 (13.7%)
Coronavirus	8 (6.1%)
Human bocavirus	3 (2.3%)

The most frequent causative agent was RSV (44.3%), followed by RV (33.6 %). Less frequently detected viruses were coronavirus (6.1%) and human bocavirus (2.3%), that were detected only as co-infective agents. Co-infection with RSV and RV was detected in 11/131 (8.4%) infants, with RSV and other than RV viruses in 29/131 (22.1%) infants, RV and other than RSV viruses in 17/131 (12.9%) infants.

#### Seasonal Distribution

The seasonal distribution of respiratory viruses in the study followed the usual patterns described before in temperate countries (23).

RV was detected throughout the year with its peaks during the autumn months of October and November. Of note, the few cases of acute bronchiolitis identified during summer were mainly caused by RV.

Influenza viruses (IFVs) showed a stable high frequency during February and March in both years and their rates were consistent with the influenza epidemics.

Parainfluenza viruses (PIVs) were scattered throughout the year. The RSV-RV dual infections were mainly detected during March in both years.

#### Machine Learning Model

##### Clustering

Following clustering analysis of recurrent wheeze episodes during 3 years of follow-up, a 2-cluster model was chosen to describe the number of wheeze exacerbations over time for each patient. Cluster 1 consisted of infants who presented a high number of wheeze episodes during follow-up and Cluster 2 consisted of infants who presented a low number of wheeze episodes during follow-up. The description of Clusters 1 and 2 in relation to the number of recurrent wheeze exacerbations over time can be seen in Supplementary Figure 1A.

Visualization of clusters in 2 principal components (PC1 and PC2) can be seen in Supplementary Figure 1B.

##### Association Between Virus Types and Clusters

**Figure 2:**
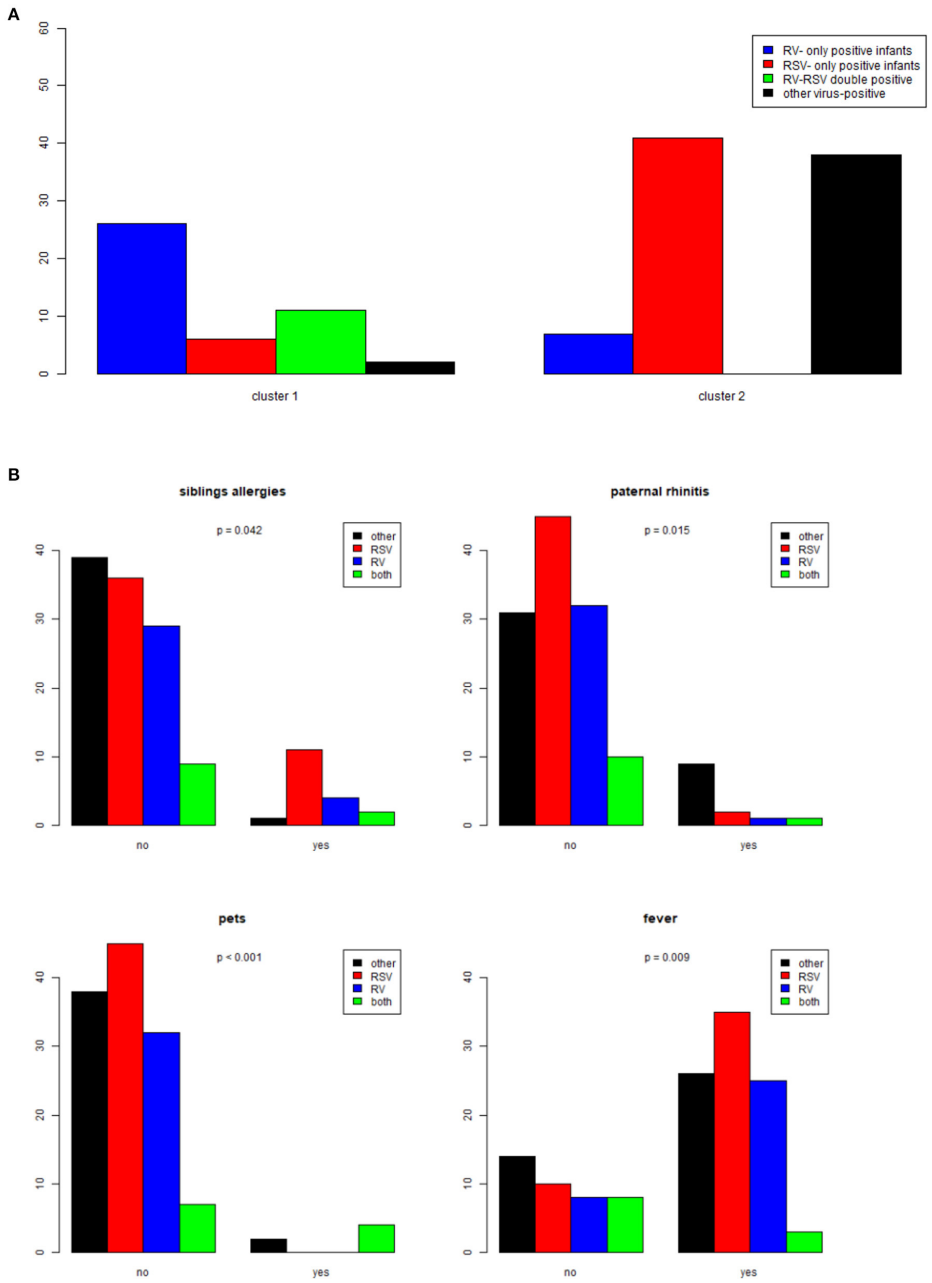
**(A)** Association between respiratory viruses detected in nasopharyngeal washes (RV-only, RSV-only, RV-RSV double positive, other virus-positive) and Clusters 1 and 2. **(B)** Graph depicting significant correlations between demographic and clinical characteristics and viruses in nasopharyngeal washes (RV-only, RSV-only, RV-RSV double positive, other virus-positive).

Detection of RV-only in nasopharyngeal washes was significantly associated with Cluster 1, while detection of RSV-only and other than RV and RSV viruses was significantly associated with Cluster 2 (Chi-squared test, *p* < 0.001) (Figure 2A).

##### Differences in Demographic and Clinical Characteristics in Infants With RV-, RSV-, RV and RSV- and Other Than RV and RSV-Induced Bronchiolitis

Univariate analysis (Chi-square tests for qualitative independent variables and Kruskal-Wallis test for quantitative independent variables) was performed with dependent variables the four groups of bronchiolitis infants based on virus detection data. Absence of history of siblings' allergies and paternal rhinitis was strongly correlated with RSV-only induced bronchiolitis (*p* = 0.042 and *p* = 0.015, respectively). Also, significantly higher number of infants with RSV-bronchiolitis were living in a household free of pets (*p* < 0.001). Fever during initial presentation was also more frequent in infants with RSV-bronchiolitis (*p* = 0.009). These correlations are depicted in Figure 2B.

**Figure 3:**
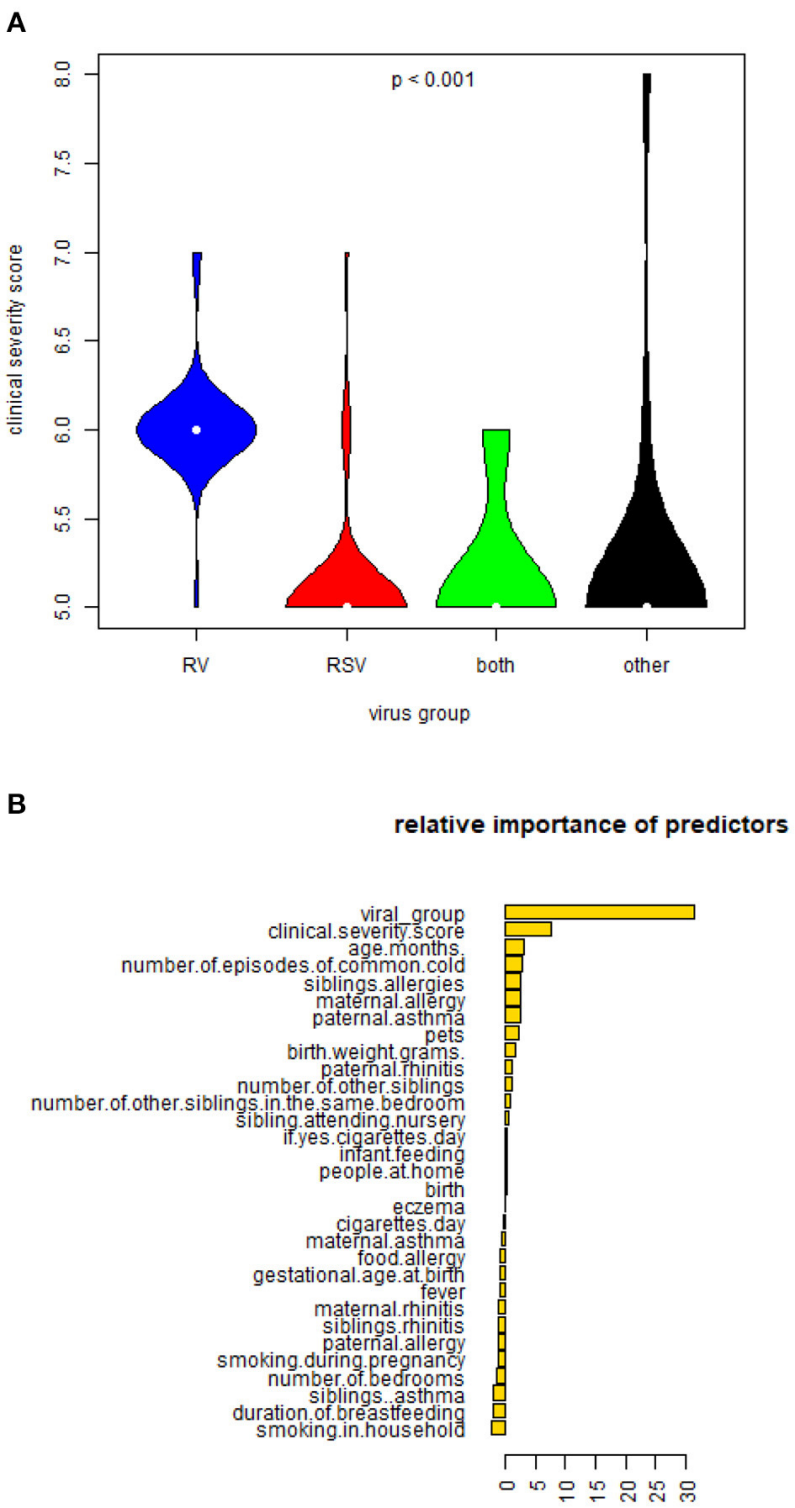
**(A)** Violin plots depicting differences in mean clinical severity score in infants hospitalized with RV-only, RSV-only, RV and RSV-double positive and other virus-positive bronchiolitis. **(B)** Figure describing the relative importance of predictors of cluster outcomes.

Infants with RV-single infections had higher severity scores when compared to those with RSV-single infections (*p* = 0.0388). Most infants with acute bronchiolitis caused by RV as a sole agent (18/24, 75%) had clinical severity score over 5, in contrast to those with RSV infection alone (11/24, 45%) or RV/RSV co-infection (5/11, 45.5%). Bonferroni pair-wise comparisons RV vs. RSV, RV vs. other viruses and RV vs. RV and RSV (*p* < 0.001). Clinical severity scores in relation to the type of triggering virus are shown in Figure 3A.

##### Predictors of Clusters

**Figure 4:**
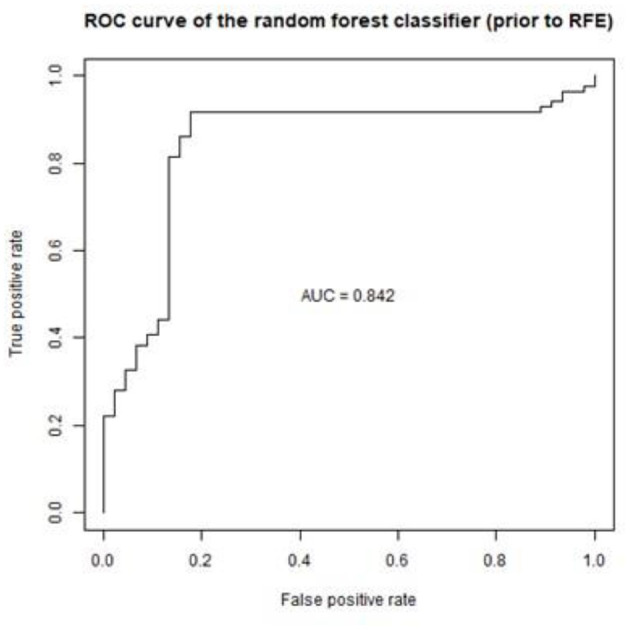
Graph depicting the ROC curve of the prediction model (sensitivity 75.56% and specificity 91.86%).

Multi-factorial analysis (with random forest classifier) guided prediction of Cluster 1 (infants who persistently presented recurrent wheeze episodes during follow-up) from the independent variables (clinical characteristics, demographics, and the viral group) with an overall error of 11.45%. The virus type was the strongest predictor of Cluster 1 (Figure 3B). Random forests classifiers use bagging (bootstrap aggregating) and feature randomness when building each individual tree to try to create an uncorrelated forest of trees (this includes adjusting for confounding between parameters utilized as predictors). A prediction model utilizing as predictors the virus type and clinical severity score could predict Cluster 1 with a calculated sensitivity of 75.56% and specificity 91.86% taking Cluster 2 (best outcome) as the base level. The graphical plot that illustrates the sensitivity and specificity of the prediction model [Receiving Operating Characteristic Curve (ROC)] can be seen in Figure 4.

Out of the 120 infants who completed the 3-year follow-up, 61 infants presented at least one episode of wheeze during the follow-up period (50.8%). Infants from the control group were not included in the cluster and the prediction models. It is of note that no control infants completing the 3-year follow-up presented wheeze episodes during the follow-up period.

## Discussion

By utilizing a machine learning approach, our study showed that in a cohort of infants hospitalized with the first episode of bronchiolitis, detection of RV and increased clinical severity on admission are strongly associated with increasing number of recurrent wheeze episodes during 3 years of follow-up. Virus type (RV) is a stronger predictor of wheeze exacerbations when compared with other clinical and non-clinical factors (including clinical severity score during admission).

Most epidemiological data in asymptomatic children come from case-control studies in children with underlying conditions or with greater risk to develop asthma. In a cohort of otherwise healthy children, it was noted that in younger than 1-year old asymptomatic infants, detection of respiratory viruses is below 10%, similar to our study, likely explained by reduced interaction with other children or adults (no nursery or school attendance) (24). Genomic profiling between asymptomatic control patients who have tested positive for RV and healthy control subjects does not differ (25). Asymptomatic control patients who tested positive in rhinovirus were followed-up and did neither develop bronchiolitis nor preschool wheeze.

One of the predictors of recurrent wheeze relapses is the clinical severity score. Clinical decision for admission at the hospital with bronchiolitis is mainly based on assessment of clinical severity (26). Therefore, infants with bronchiolitis who are severe enough to require admission, are more likely to present recurrent wheeze episodes during the first years of life (27). This observation has been reported in other cohorts as well (9). Data from the Multicenter Airway Research Collaboration-35 Study (MARC-35) show that increased genomic load of other than Severe Acute Respiratory Syndrome coronavirus type 2 (SARS-CoV-2) coronaviruses detected in nasopharyngeal samples, is associated with increased severity of bronchiolitis and need for escalation of management (28). Our cohort shows that infants with RV-induced bronchiolitis had more severe clinical presentation as compared to infants with other than RV-induce bronchiolitis. Genomic viral loads were not measured as there was no infant in the cohort who required escalation of respiratory management.

There are several cohort studies that have associated rhinovirus with recurrent wheeze and asthma development (29–31). Data from a prospective, 17-center US cohort study of 921 infants hospitalized with severe bronchiolitis show that the cluster of infants who have already suffered with bronchiolitis, have eczema and get hospitalized with RV-induced illness, present higher number of wheeze relapses (17). Our study shows similar pattern in a cohort of infants hospitalized with the first episode of bronchiolitis. The associations with RV and wheeze relapses has also been confirmed in a cohort of infants who are at risk to develop asthma, where the RV-induced first episode of bronchiolitis was shown to be associated with recurrent episodes of wheeze during the first years of life (16). Therefore, both birth cohort and bronchiolitis cohort studies highlight RV-induced bronchiolitis hospitalization in infancy as a marker of susceptibility to recurrent wheeze development.

These observations are challenging the traditional knowledge in general pediatricians around bronchiolitis etiology, severity, and outcomes. It is however impressive that this thinking has not changed, mainly because training of young pediatricians still focuses on repeating the dogma that severe bronchiolitis is mainly caused by RSV and RSV severe bronchiolitis is the strongest predictor of recurrent wheeze and asthma development. There is no doubt that this notion was built on evidence coming from series of observational cohorts (32–35). RSV has been highly associated with wheezing and asthma development (32–41) in studies where comparisons have been made with healthy infants, but, when attempting to compare with infants who get hospitalized with other than RSV viruses, RSV has failed to be shown as a significant link to wheeze and asthma (42, 43). More importantly, apart from Sigurs' studies (41), most of the studies reporting an effect of RSV on the development of a recurrent wheeze phenotype, cannot confirm allergic sensitization or atopic predisposition, that, for some clinicians, are strong predictors of recurrent wheeze persistence.

This could explain differences in the use of respiratory virus testing. In the UK, respiratory virus testing is used mainly for hospitalized infants with bronchiolitis and, frequently, it is limited on a rapid RSV test. The test is used mainly as a guide for isolating bronchiolitis patients, rather than identifying which patients require more frequent clinical review post hospital discharge. However, a recent population-based cohort study showed that one in five infants with bronchiolitis will have at least a hospital admission within the first 5 years of life associated with wheeze, asthma or a lower respiratory tract infection (27).

The strengths of this study are the inclusion of a milder severity spectrum of hospitalized infants with bronchiolitis. Also, the recruitment took place throughout the year and not only in the RSV-peak period, the recruitment of control patients and regular phone follow-up was completed in most patients and controls. This study also defined “clusters” of wheeze exacerbations replaced absolute numbers of wheeze exacerbations. These clusters have been suggested to describe trajectories of “recurrence of wheeze.” This is important as most studies assessing wheeze exacerbations as outcome are based on caregivers' descriptions, which frequently under- or overestimate the absolute number of wheeze exacerbations.

This study has three main limitations. The first limitation is focused on the small number of patients. Although the number of recruited patients was small, there was a small drop in follow-up until the age of 3 years in the bronchiolitis group (8.3% drop in follow-up). With the application of the “random forest methodology,” we aimed to obtain a better prediction performance in a small group of patients. Both the clustering and the multi-factorial analysis based on the clustering did not utilize any data from the control group. As the incidence of wheeze exacerbations in the control group was small, including these in the model would make the predictive probability severely overestimated for future patients (44). The second limitation focuses on the assessment of wheeze exacerbations. Telephone interviews were used instead of healthcare activity data. The electronic system in Greece was not developed so that attendances for wheeze exacerbations could be screened through healthcare activity data. However, even in countries as the UK, with advanced healthcare activity systems, getting accurate information for hospital attendances with wheeze exacerbations would be tricky. Most parents do not always attend one hospital. Sometimes children are reviewed in primary care, and the integration of data between primary care and hospitals is now developing. In summary, assessing wheeze exacerbations through telephone interviews contains the risk of over-reporting wheeze, but the follow-up was taking place every 6 months, therefore this would be a recent (easy to remember) event for parents. The third limitation focuses on the assessment of allergic sensitization. However, we assessed evidence of family history of allergies during study recruitment. The assessment of allergic sensitization and the incorporation of these data on a prediction model algorithm for infants hospitalized with bronchiolitis could be explored in future studies. This study did not assess specific rhinovirus subtypes. Cohort studies have shown that rhinovirus-C has been associated with higher risk of developing recurrent wheeze during the first years of life (45). In a future validation study for this prediction model, it will be important to include virus subtypes.

## Conclusion

The virus type (rhinovirus) detected in nasopharyngeal washes of infants hospitalized with the first episode of bronchiolitis is a sensitive predictor of recurrent wheeze trajectory during a 3-year follow up period. The external validation of this model in a birth cohort including hospitalized and non-hospitalized infants with severe bronchiolitis will help generalize these findings.

## Data Availability Statement

The raw data supporting the conclusions of this article will be made available by the authors, without undue reservation.

## Ethics Statement

The studies involving human participants were reviewed and approved by Ethics Approval Athens 29-10-2015. Ref No. 1446/22-10-2015. Paidon and Aglaia Kiriakou Hospital. Written informed consent to participate in this study was provided by the participants' legal guardian/next of kin.

## Author Contributions

NP: designed, supervised the study, and the funding applications for the study and reviewed the manuscript. VP: supervised the study and the funding applications for the study and reviewed the manuscript. MT: supervised participants' recruitment and reviewed the manuscript. SJ and PX: reviewed the manuscript and offered expert input. MM: helped with the design of the fire plot. ST and SM: helped with molecular testing and reviewed the manuscript. JL: performed the statistical analysis. HM: completed the participants' recruitment, the follow-up, the descriptive statistics, and wrote the manuscript. All authors contributed to the article and approved the submitted version.

## Funding

This study has received funding from GSK GlaxoSmithKline, GR (2008), ELKE code 70/3/9478. Differential association of viral agents in wheeze exacerbations in hospitalized children. SJ is the Asthma UK Clinical Chair (grant CH11SJ) and a National Institute of Health Research (NIHR) Emeritus Senior Investigator and was funded in part by European Research Council Advanced Grant 788575. This research was supported by the NIHR Imperial Biomedical Research Centre. The views expressed are those of the author(s) and not necessarily those of the NIHR or the Department of Health and Social Care.

## Conflict of Interest

NP reports personal fees from Novartis, personal fees from Nutricia, personal fees from HAL, personal fees from MENARINI/FAES FARMA, personal fees from SANOFI, personal fees from MYLAN/MEDA, personal fees from BIOMAY, personal fees from AstraZeneca, personal fees from GSK, personal fees from MSD, personal fees from ASIT BIOTECH, personal fees from Boehringer Ingelheim, grants from Gerolymatos International SA, grants from Capricare, outside the submitted work. The remaining authors declare that the research was conducted in the absence of any commercial or financial relationships that could be construed as a potential conflict of interest.

## Publisher's Note

All claims expressed in this article are solely those of the authors and do not necessarily represent those of their affiliated organizations, or those of the publisher, the editors and the reviewers. Any product that may be evaluated in this article, or claim that may be made by its manufacturer, is not guaranteed or endorsed by the publisher.
