# Supplementary material for: Recurrent Wheeze Exacerbations Following Acute Bronchiolitis—A Machine Learning Approach
Source: Front Allergy. 2021 Nov 2;2:728389. doi: 10.3389/falgy.2021.728389 (PMC8974688; doi:10.3389/falgy.2021.728389)

Recurrent wheeze exacerbations following acute bronchiolitis – a machine learning approach

Makrinioti H. ^1,2†*^, Maggina P^.3†^, Lakoumentas J. ^3^, Xepapadaki P. ^3^, Taka S. ^3^, Megremis S.^4^, Manioudaki M. ^3^, Johnston SL.^5^, Tsolia M.^6^, Papaevangelou V.^7†^, Papadopoulos NG ^3,4†^

Figure Ia. Average number of wheeze episodes over 36 months (3 years) of follow-up in Clusters 1 and 2 of infants with bronchiolitis


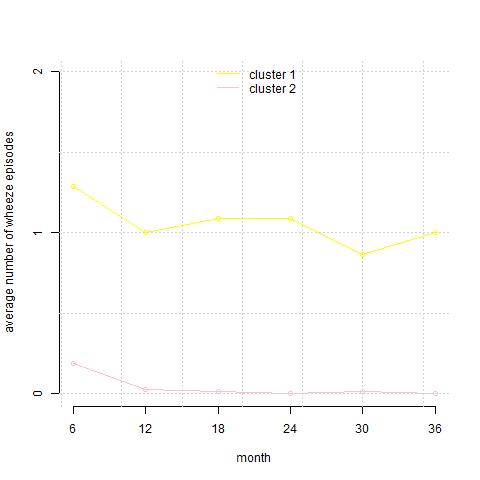


Figure Ib. PCA biplot visualizing Cluster 1 (1,1,1,1,1,1) and Cluster 2 (2,2,2,2,2,2)


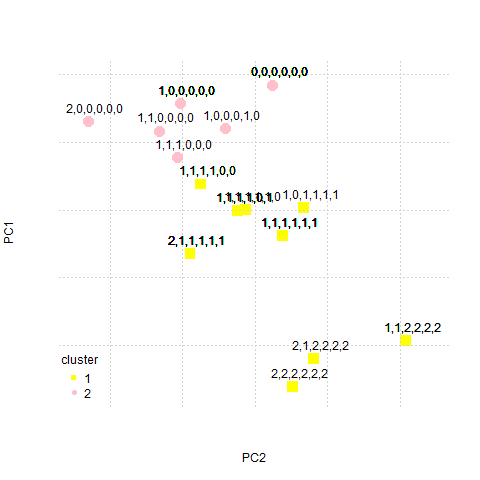

Supplement: Supplementary file 1 [file Data_Sheet_1.docx]
